# Expression of Arabidopsis *WEE1* in tobacco induces unexpected morphological and developmental changes

**DOI:** 10.1038/s41598-019-45015-3

**Published:** 2019-06-18

**Authors:** Ilario Siciliano, Anne Lentz Grønlund, Hana Ševčíková, Natasha D. Spadafora, Golnaz Rafiei, Dennis Francis, Robert J. Herbert, M. Beatrice Bitonti, Hilary J. Rogers, Helena Lipavská

**Affiliations:** 1 School of Biosciences, Cardiff University, Sir Martin Evans Building, Museum Avenue, Cardiff, CF10 3AT UK; 2 Department of Experimental Plant Biology, Charles University, Faculty of Science, Viničná 5, 128 43, Praha 2, Czech Republic; 3 School of Science and the Environment, University of Worcester, Henwick Grove, Worcester, WR2 6AJ UK; 4 Department of Biology, Ecology and Earth Sciences, University of Calabria, Arcavacata di Rende, Cosenza, Italy

**Keywords:** Plant signalling, Plant cell cycle

## Abstract

WEE1 regulates the cell cycle by inactivating cyclin dependent protein kinases (CDKs) via phosphorylation. In yeast and animal cells, CDC25 phosphatase dephosphorylates the CDK releasing cells into mitosis, but in plants, its role is less clear. Expression of fission yeast CDC25 (*Spcdc25*) in tobacco results in small cell size, premature flowering and increased shoot morphogenetic capacity in culture. When *Arath;WEE1* is over-expressed in Arabidopsis, root apical meristem cell size increases, and morphogenetic capacity of cultured hypocotyls is reduced. However expression of *Arath;WEE1* in tobacco plants resulted in precocious flowering and increased shoot morphogenesis of stem explants, and in BY2 cultures cell size was reduced. This phenotype is similar to expression of *Spcdc25* and is consistent with a dominant negative effect on WEE1 action. Consistent with this putative mechanism, WEE1 protein levels fell and CDKB levels rose prematurely, coinciding with early mitosis. The phenotype is not due to sense-mediated silencing of WEE1, as overall levels of WEE1 transcript were not reduced in BY2 lines expressing *Arath;WEE1*. However the pattern of native WEE1 transcript accumulation through the cell cycle was altered by *Arath;WEE1* expression, suggesting feedback inhibition of native WEE1 transcription.

## Introduction

The eukaryotic cell cycle is a conserved phosphorylation cascade in which key substrates require phosphorylation or dephosphorylation prior to the next step in the cell cycle. At the G1/S and G2/M transitions, major phosphoregulation occurs, catalysed by cyclin-dependent protein kinases (CDKs) that are largely conserved in unrelated species^1^. However, unlike animal cell cycles, in plants two CDKs, A and B regulate the transition from G2 to mitosis^2,3^. CDKA is closely related to the ancestral *cdc2* of fission yeast and is able to complement temperature sensitive *cdc2*^*-*^ mutants. In tobacco BY2 cells, CDKA transcript^4^ and protein^5^ levels are constant throughout the cell cycle, whereas activity peaks at S/G2^5^. The CDKB family (1;1, 1;2, 2;1 and 2;2) is unique with the highly conserved PSTAIRE domain of cdc2 (and CDKA) altered to PPTLARE/PPTLRE^3^. Also, Arabidopsis CDKB genes are unable to complement *cdc2*^-^/*cdc28*^6^. CDKB transcripts^5^, protein and activity all peak at G2/M^4^.

In fission yeast, Wee1 and Mik1 kinases phosphorylate Tyr15 of the CDK to inactivate it and prevent entry into mitosis^7,8^. When Wee1 is over-expressed in fission yeast (*Schizosaccharomyces pombe*) cells arrest in G2 resulting in highly elongated cells^7^. Conversely, the phosphatase, Cdc25, dephosphorylates the same tyrosine residue activating the CDK^9^. The role of CDC25 in the plant cell cycle is less clear. A truncated version of the yeast CDC25 gene containing only the catalytic domain is present in the Arabidopsis genome^10^ and can induce a short cell length when expressed in fission yeast^11^. However, its role in the plant cell cycle seems to be limited to the DNA damage replication checkpoint^12^ as the plants grow and develop normally. Thus perturbation of *Arath;CDC25* expression in Arabidopsis resulted in hypersensitivity to hydroxyurea while over-expression resulted in tolerance compared to wild type. Moreover, although *Arath;*CDC25 has phosphatase activity^10^ it also has arsenate reductase activity^13^ suggesting that in plants CDC25 may have additional roles outside of the cell cycle.

Given the uncertainties around plant CDC25, fission yeast CDC25 (*Spcdc25*) was expressed in plant cells to study the effects of CDK de-phosphorylation^14^. Expression of the fission yeast CDC25 gene in both tobacco^15^ and Arabidopsis^16^, resulted in phenotypes that are consistent with its action in dephosphorylating and activating CDK. Expression of *Spcdc25* in tobacco BY2 cells resulted in a reduced mitotic cell size and a reduction in the length of the G2 phase^17^. Moreover, in these cells, cytokinin levels were greatly reduced and the cells were insensitive to the cytokinin biosynthetic inhibitor, lovastatin indicating a link between CDK de-phosphorylation and cytokinin signalling. In addition, *Spcdc25* expression in tobacco cell suspension cultures altered carbohydrate status resulting in an increase of starch and soluble sugars and a higher sucrose:hexose ratio. These changes are inducible in WT by cytokinin treatment, thus, *Spcdc25* expression in tobacco had a cytokinin-like effect^18^. In whole plants, this cytokinin-independent phenotype was supported by an ability of *Spcdc25* expressing stem explants to produce shoots in the absence of exogenous cytokinin^19^. Consistent results were obtained in Arabidopsis plants expressing *Spcdc25*^16^, which showed a reduction in primary root length and increased production of lateral roots. Another effect of *Spcdc25* expression in tobacco was precocious flowering with a dramatic reduction in both the time to flowering, and the number of leaves and nodes formed prior to flowering^20^. Moreover, study of flowering of tobacco nodal stem segments *in vitro* revealed that the typical acropetal flowering gradient in WT plants did not occur in the *Spcdc25* transgenic plants^21^. However when *Spcdc25* was expressed in Arabidopsis, flowering time was not affected (Rogers and Francis lab. unpublished data).

Where the plant cell cycle diverges quite dramatically from other eukaryotes, is that Arabidopsis mutants deficient in WEE1 kinase grow and develop normally although they are hypersensitive to DNA replication inhibitors such as hydroxyurea^10,22^. However, the role for WEE1 in plants is not restricted to the DNA replication checkpoint. WEE1 regulates CDK activity in a cell cycle dependent manner with a drop in WEE1 activity at the G2/M transition^23^ and in both tobacco BY2 cells and in Arabidopsis roots, WEE1 protein is removed as cells enter mitosis via the 26 S proteasome.

Cultured hypocotyls of *Arabidopsis wee1-1* mutants showed increased morphogenetic capacity, and *wee1-1* seedlings produced more lateral roots per millimetre of primary root^24^. Conversely over-expression of *Arath;WEE1* in *Arabidopsis* repressed the morphogenetic capacity of hypocotyls in culture and primary roots of these transgenic plants were shorter with less lateral roots than in the wild type. In *Arabidopsis* over-expressors of *Arath;WEE1* also displayed larger cell size and slower cell doubling time in the root apical meristem. In tobacco BY2 cells, expression of tomato WEE1 (*Solly;WEE1*) resulted in increased overall WEE1 protein, reduction in CDKA histone H1 kinase activity and an increase in phosphorylated CDKA^25^. This was accompanied by an increase in cell size and a delay in the G2/M transition in synchronised cells. However, surprisingly, when *Arath;WEE1* was expressed in tobacco BY2 cells, there was a shortening of G2^23^. This was reversed by co-expression of the F-box protein SKP1 INTERACTING PARTNER 1 (SKIP1), which interacted with WEE1, presumably removing it through the 26 S proteasome.

Data are presented here showing that the anomalous effects of *Arath;WEE1* expression in tobacco cells are mirrored by effects on the development of whole plants, and is consistent with a perturbation of the native tobacco WEE1, creating a dominant-negative-like effect.

## Results

### *Arath;WEE1* expression in tobacco plants results in premature flowering, altered root system growth and spontaneous shoot formation in culture

**Figure 1:**
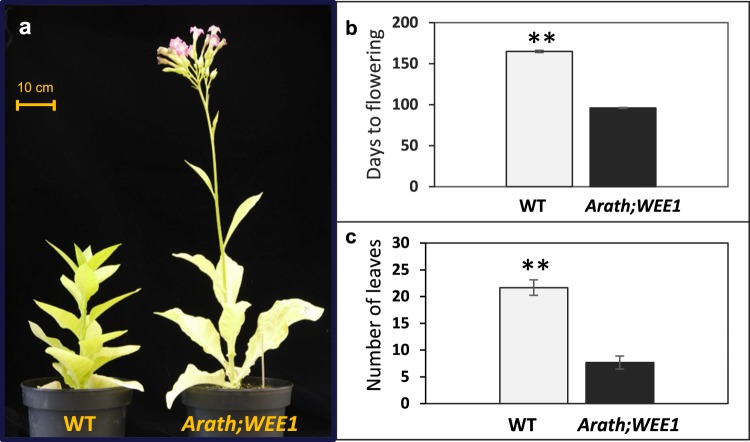
Expression of *Arath;WEE1* in tobacco plants changed growth habit and onset of flowering. Tobacco plants (WT) and NT-Arath;Wee1#8: (**a**) after 100 days of growth; (**b**) number of days and (**c**) number of leaves over 10 cm in length, at flowering (n = 6 ± SE; **P < 0.01).

Constitutive *Arath;WEE1* expression in tobacco (Fig. S1) caused significant changes in plant development and led to premature flowering (Fig. 1a). WT plants grown in a growth chamber took approximately 150 days to flower (production of first visible bud) from day of sowing, whereas the *Arath;WEE1* –expressing transgenic plants (NT-Arath;Wee1#8 and #2) flowered significantly earlier, after about 100 days (Fig. 1b). Moreover, WT plants flowered when they had produced more than 20 leaves longer than 10 cm, while transgenic plants expressing *Arath;WEE1* formed only around seven leaves of this size before they started to flower (Fig. 1c).

**Figure 2:**
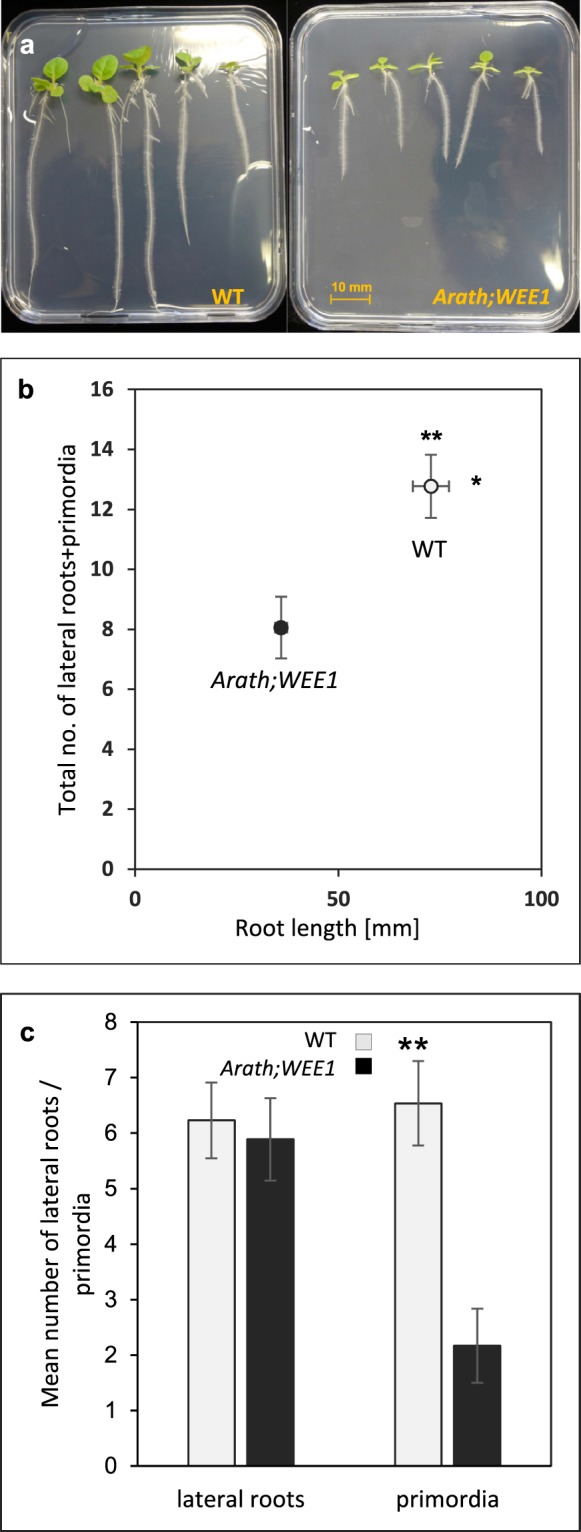
Tobacco root development is affected by expression of *Arath;WEE1*. (**a**) Root growth after 21 days, of wild type (WT) and NT-Arath;Wee1#8 on MS medium. (**b**) The relationship between mean total number of lateral roots and lateral root primordia and mean primary root length for 21-d-old seedlings. (**c**) Mean number of lateral roots and primordia (n = 18 ± SE; *P < 0.05; **P < 0.01).

Expression of *Arath;WEE1* in tobacco plants also affected root development. NT-Arath;Wee1#8 plants had a significantly shorter primary root and when they were considered together, they had significantly fewer lateral roots + root primordia (Fig. 2a,b). However, when considered separately, there was no difference in the number of lateral roots between WT and *Arath;WEE1*-expressing plants, while  there were fewer primordia in the transgenic line (Fig. 2c). This indicates that *Arath;WEE1*-expressing plants form less primordia with better capacity for outgrowth into fully grown lateral roots.

**Figure 3:**
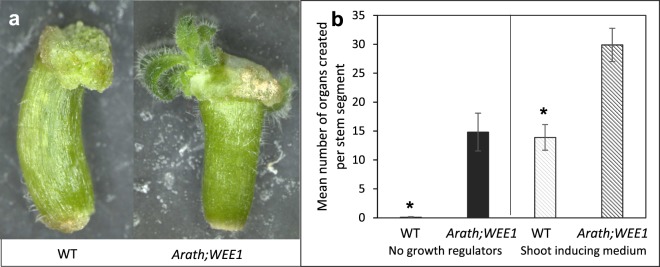
*De novo* shoot formation from tobacco stem explants is stimulated by expression of *Arath;WEE1*. (**a**) Shoot formation after growth of wild type (WT) and NT-Arath;Wee1#8 21 days on MS medium; (**b**) organogenesis on medium without addition of plant growth regulators and shoot inducing medium (n = 12 ± SE; * *P* < 0.05).

Further effects of expressing *Arath;WEE1* in tobacco plants were seen in culture. When growing on standard cultivation media without any growth regulators, 1 cm long stem cuttings from *Arath;WEE1*-expressing plants formed on average 15 new shoots compared to WT cuttings that formed only callus (Fig. 3). NT-Arath;Wee1#8 tobacco stem segments cultivated on shoot induction medium also showed significantly greater capacity to form new shoots, producing 30, on average, from each stem cutting, while WT cuttings formed on average only 14.

### *Arath;WEE1* expression in tobacco BY2 cells resulted in a reduction in mitotic cell size and a shortening of G2

**Figure 4:**
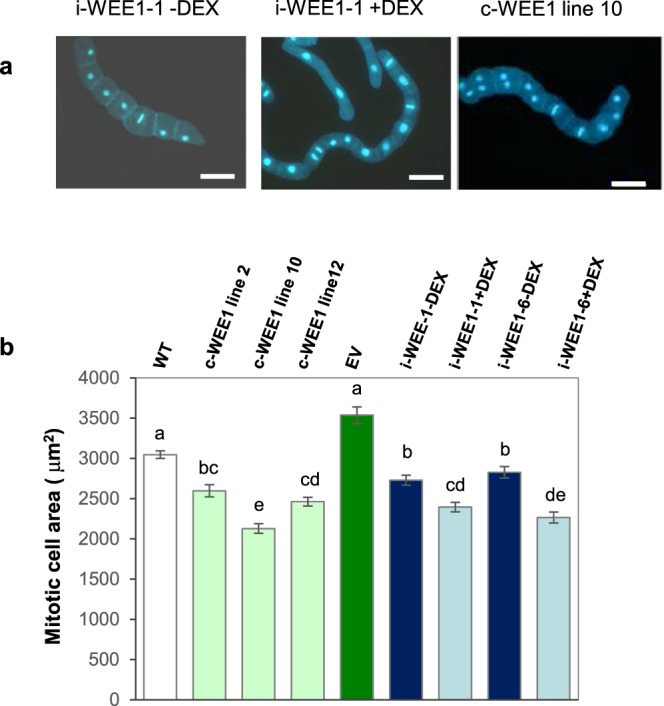
Expression of *Arath;WEE1* in BY2 cells results in a small mitotic cell size. (**a**) Mitotic cells in i-WEE1-1 ± DEX (bar = 100 µm), (**b**) Mean mitotic cell area (µm^2^ ± S.E.) in wild type (WT), constitutively ‘c’ expressing WEE1 lines: 2, 10 and 12 compared with empty vector (EV), and in inducible lines ‘i’ 1 and 6 ± DEX (n = 300). Lower case letters indicate significant differences based on a Kruskall Wallis test followed by a Dunn’s test (*P* < 0.05).

**Figure 5:**
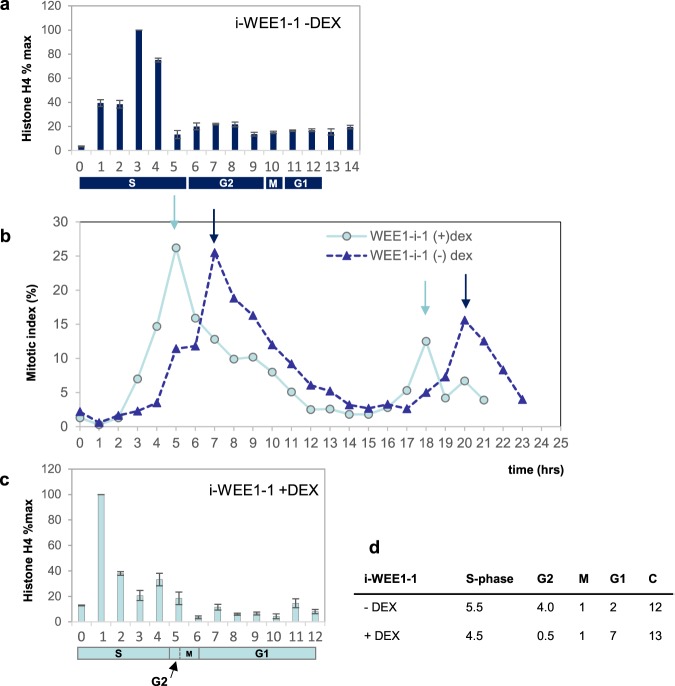
Inducible *Arath;WEE1* expressing BY2 cells have a short G2. (**b**) Changes in the mitotic index following synchronisation with and removal of aphidicolin in i-WEE1−1 -DEX (dark dashed lines) or +DEX (light solid lines). The pairs of dark and light arrows mark the cell cycle times for each line/treatment: BY2 cells blocked in late G1 and S-phase by aphidicolin and then released following drug removal show an initial rise in the curve when cells trapped at the end of S-phase during the aphidicolin block, are the first to traverse G2 and enter mitosis following removal of the block. Since the first peak is when the bulk of synchronised cells enter mitosis, this point in time minus S-phase is an alternative measure of G2. Either way, G2 is less than 1 hour in the +DEX treatment, and 4 h −DEX (representative data from replicate experiments). Above and below the cell cycle plots, are mean expression profiles of histone H4 as percentages of maximum expression (±SD) without (**a**) and with (**c**) DEX used to calculate S-phase (4.5 h +DEX, 5.5 h −DEX). SEM was <3% throughout; n = 3. The duration of M-phase was calculated from the average mitotic index for each treatment (M) using formulae developed by Nachtwey and Cameron (1968) which account for exponential growth: dM = C/ln2 ×  ln (M + 1). G1 is calculated by difference. All phase durations are in hours.

When *Arath;WEE1* was expressed constitutively in three independent BY2 cell lines (c-WEE1 lines 2, 10 and 12; Fig. S2a), significant reductions in mitotic cell size (*P* < 0.05) were detected compared with the empty vector line (EV) or with WT (Fig. 4a,b). A similar result was obtained when BY2 cells were transformed with *Arath;WEE1* using an inducible vector and expression induced by DEX in two independent lines (i-WEE-1 and i-WEE-6; Fig. S2b–d). A consistent effect on cell size was not seen when DEX was added to BY2 cells transformed with the empty pTA002 vector (Fig. S3). The effect of *Arath;WEE1* on cell size correlated with effects on cell cycle progression. When expression of *Arath;WEE1* was induced in line i-WEE-1 that was synchronised using aphidicolin, the mitotic index curve rose sooner (1–2 h) and peaked earlier (4–5 h) compared with the minus DEX control in which expression of *Arath;WEE1* was not induced (Fig. 5b). These curves are consistent with a shortened G2 when *Arath;WEE1* was expressed, as shown by histone H4 profiles used to measure the duration of S-phase, which was 5 to 6 h −DEX (Fig. 5a) and 4 to 5 h in the +DEX treatment (Fig. 5c). The interval between peaks (indicated by arrows in Fig. 5b) spans a cell cycle time of 13 and 12 h in the −DEX and +DEX treatments, respectively. Hence following induction of *Arath;WEE1* expression, the major effect on the cell cycle was an 8-fold shortening of G2 compensated by a 3-fold lengthening of G1 (Fig. 5d).

### Total WEE1 protein levels increased on expression of *Arath;WEE1*, and the pattern of WEE1 protein levels and activity were altered in synchronised cultures

**Figure 6:**
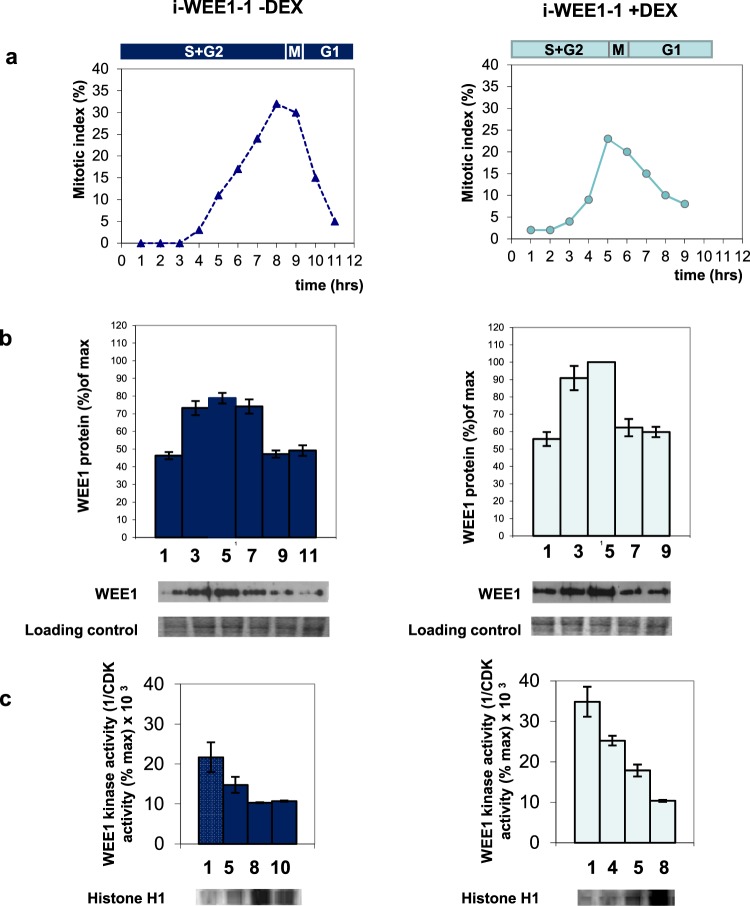
WEE1 protein level and kinase activity in a synchronized transgenic *N. tabacum* BY2 cell culture carrying pTA7002 *Arath;WEE1*. Left: −DEX. Right: +DEX. (**a**) Mitotic index profile was calculated as the sum of prophase, anaphase, metaphase, and telophase mitotic figures as a percentage of minimum 300 cells. The corresponding cell cycle phases are shown above the mitotic index graph. (**b**) Immunodetection of WEE1 protein (Total WEE1 (Nicta;WEE1 + Arath;WEE1) for +DEX). Proteins were extracted from synchronized  samples and subjected to Western blotting using a Nicta;WEE1 antibody. Histogram displays mean (±SE) WEE1 expression levels (n = 3). A representative Western blot and a Coomassie stain loading control are shown below the histogram. (**c**) WEE1 kinase inhibition assay. Histogram displays the mean (±SE) WEE1 kinase activity levels (n = 2). The incorporation of ^32^P was assayed by quantification of the bands on the autoradiograph and WEE1 kinase activity was expressed as the reciprocal of CDK activity (1/CDK activity (% max) × 10^3^). A representative autoradiograph is shown below histogram.

To establish the mechanism of the cell cycle changes, effects on the timing of changes in WEE1 protein during the cell cycle were investigated. In synchronised BY2 cell lines transformed with an inducible *Arath;WEE1* construct, without addition of DEX, total WEE1 protein levels increased through S/G2, however, as the majority of the cells entered mitosis (after 7 h), the WEE1 protein level decreased (Figs 6a,b; S6). When expression of *Arath;WEE1* was induced by addition of DEX, WEE1 protein levels increased during S/G2 and again fell rapidly as cells entered mitosis (after 5 h). Hence, changes in WEE1 protein through the cell cycle followed the altered timing of mitosis in the induced cells.

A WEE1 kinase inhibition assay was used to investigate whether the WEE1 protein levels correlated with changes in the timing of WEE1 kinase activity. WEE1 activity was measured as the inhibitory action of immunoprecipitated WEE1 protein on CDK activity, using histone H1 as substrate (Fig. 6c). Sampling times were selected to coincide with early S phase and G2/M in both ±DEX. Without induction of *Arath;WEE1* by addition of DEX, WEE1 kinase activity was maximal in early S phase and decreased by 31% in late G2 reaching a minimum during mitosis, consistent with the observed decrease in WEE1 protein level. In induced cultures, the WEE1 kinase activity was again maximal in early S phase and decreased by 29% in G2 and by a further 28% when the mitotic index peaked. Thus, WEE1 kinase activity also followed WEE1 protein levels and the altered timing of the mitotic peak.

### Expression of *Arath;WEE1* resulted in a premature increase in Nicta;CDKB1 activity

**Figure 7:**
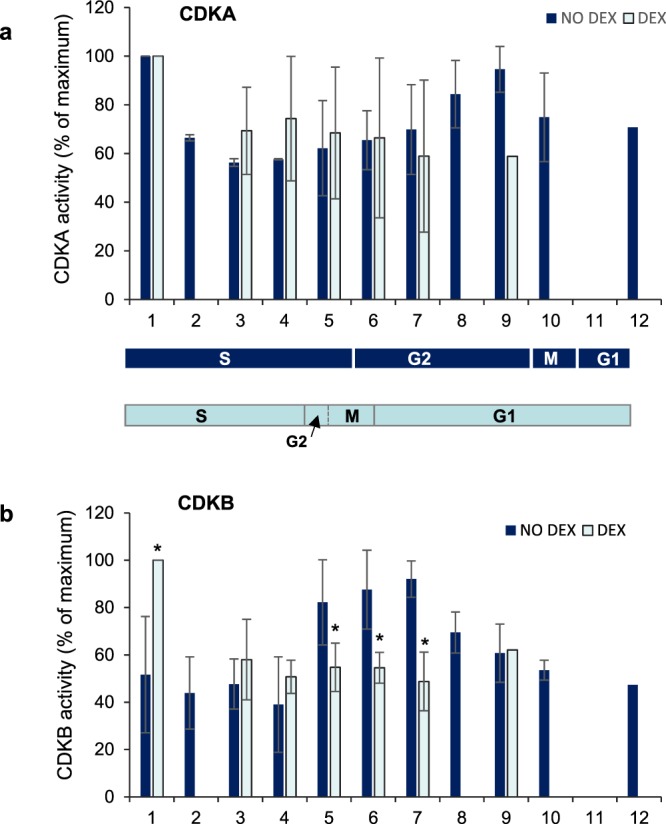
Nicta;CDKB1 kinase activity is altered when *Arath;WEE1* is expressed in BY2 cells. Mean histone H1 kinase activities in i-WEE1-1 (±SD) of (**a**) CDKA (**b**) CDKB, ±DEX. Immunoprecipitates in triplicate experiments were quantified as a percentage of maximum (n = 3) for each treatment. Protein extracts for these assays were sampled from the same experiment used to generate mitotic index curves in Fig. 2 and the phase durations are carried over between plots. Stars indicate significant differences between ±DEX at each time point based on a Kruskal Wallis test followed by a Dunn’s test, P < 0.05; ns = non significant.

A logical hypothesis is that premature cell division would require early increases in CDK activity, which would drive cells into early mitoses. This hypothesis was tested by measuring kinase activity of both Nicta;CDKA;1 (referred to here, as CDKA) and Nicta;CDKB;1 (referred to here as CDKB) in the inducible *Arath;WEE1* line 1 with and without DEX induction. CDKA activity was relatively constant regardless of the addition of DEX (Figs 7a; S8). However DEX-induction of *Arath;WEE1* resulted in a significant increase in CDKB activity, compared to uninduced cells 1 h following release of the cells from aphidicolin when both induced and uninduced cells were in early S phase. In addition induced cells showed a significant reduction in CDKB activity at 5–7 h following aphidicolin release. At this point the +DEX treated cells were at G2/M, while the uninduced cells were only at S/G2 (Figs 7b; S8). Thus the induction of *Arath;WEE1* resulted in an earlier peak in CDKB activity consistent with the earlier mitotic peak.

### *Arath;WEE1* perturbed the pattern of *Nicta;WEE1* expression in synchronously dividing cells

Premature entry into mitosis at a reduced cell size could be regulated at the transcriptional, level. Expression of *Nicta;WEE1* in exponential phase BY2 cell cultures carrying the *Arath;WEE1* inducible construct following DEX induction, was compared with *Nicta;WEE1* expression in exponential phase WT BY2 cell cultures (Fig. S2e). There was a small decrease in *Nicta;WEE1* expression when *Arath;WEE1* was induced compared to WT expression, but this is unlikely to be sufficient to explain the cellular, protein and kinase changes seen in the *Arath;WEE1-*expressing cell lines. Similarly there was no significant change in the total WEE1 transcripts (*Nicta;WEE1* + *Arath;WEE1*) when *Arath;WEE1* expression was induced by DEX in BY2 cells carrying the inducible construct compared to exponential phase WT BY2 cell cultures (Fig. S2f).

**Figure 8:**
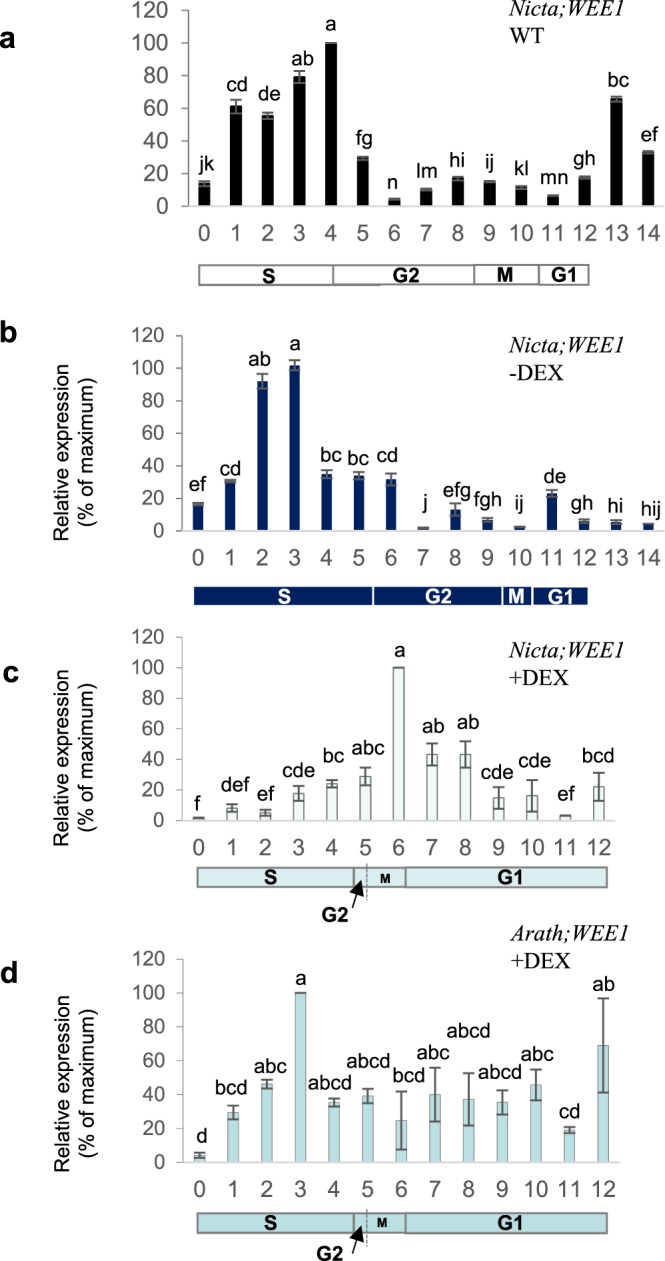
Expression of *Arath;WEE1* disrupts the *Nicta;WEE1* expression profile during the cell cycle of synchronized wild type BY2 cells following release from aphidicolin. RT-PCR of RNA extracted from (**a**) synchronised WT BY2 cells, (**b**) i-WEE1-1 −DEX, (**c** and **d**) i-WEE1-1 +DEX; using primers for *Nicta;WEE1* (**a**–**c**) or *Arath;WEE1* (**d**) (normalised to 18S rRNA, mean ± SD.; n = 3). Below the histogram is the duration of the BY2 cell cycle phases derived from the mitotic index (Fig. 5). Different lettering is based on a Kruskal Wallis test followed by a Dunn’s test, P < 0.05.

However comparing *Nicta;WEE1* expression ±DEX in synchronised cell lines, clear differences in the timing of *Nicta;WEE1* expression were evident. In WT BY2 cells and in the uninduced *Arath;WEE1* line, expression of *Nicta;WEE1* peaked in mid S-phase (Fig. 8a,b). However, when *Arath;WEE1* was induced, the pattern of *Nicta;WEE1* expression was perturbed so that the peak of its expression was shifted into mitosis/early G1 (Fig. 5c). Following induction, *Arath;WEE1* was expressed more constantly through the cell cycle than *Nicta;WEE1*, as *Arath;WEE1* expression was regulated by the 35S promoter, but significant peaks in expression were still seen in S phase and late G1 (Fig. 5d).

### Over-expression of *Nicta;WEE1* in BY2 cells did not lead to a small mitotic size phenotype

**Figure 9:**
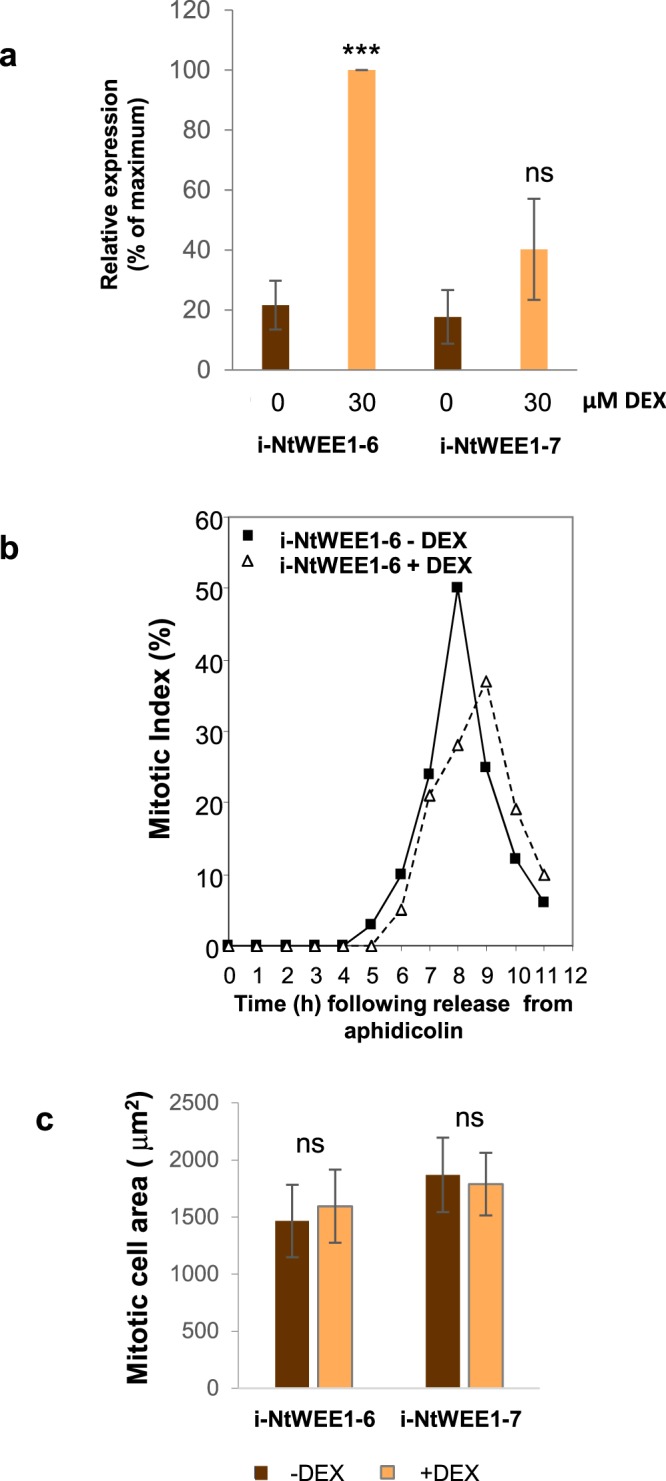
*Nicta;WEE1* over expression does not lead to a small mitotic size phenotype. (**a**) Expression levels of *Nicta;WEE1* in the presence or absence of dexamethasone in two independent transgenic lines of BY2 cells at 0 or 30 µM DEX (n = 3; +SD); ***P < 0.001, ns = non significant based on an ANOVA test followed by a Tukey’s test. (**b**) Mitotic indices following synchronisation of i-NtWEE1–6 with aphidicolin ± DEX. (n = 48); (**c**) mitotic cell area i-NtWEE1–6 and i-NtWEE1–7; n ≥ 29; +SD; ns = non significant difference between induced and uninduced area by Student’s t test (line 6) and Kruskal Wallis followed by a Dunn’s test (line 7).

To test whether the effect of *Arath;WEE1* expression in BY2 cells was a general effect of excess WEE1 expression, or whether it was specific to *Arath;WEE1*, *Nicta;WEE1* was over-expressed in BY2 cells using the same DEX inducible system in two independent lines (Fig. 9a). However when the BY2 cells were synchronised with aphidicolin, and the *Nicta;WEE1* expression was induced with DEX the mitotic peak was not anticipated as was found when *Arath;WEE1* expression was induced, in fact there was a very slight delay in mitosis (Fig. 9b). Mitotic cell area was also unaffected by over-expression of *Nicta;WEE1* in BY2 cells (Fig. 9c).

## Discussion

The flowering phenotype seen in the *Arath;WEE1* tobacco plants shows strong similarities to the phenotype seen when *Spcdc25* was expressed in tobacco^14,20,21^. The reduction in time to flowering (a 1.5 fold reduction) and number of leaves produced before flowering (a 2.8 fold reduction) was almost identical. However in contrast to tobacco plants expressing *Spcdc25*, expression of *Arath;WEE1* in tobacco plants did not result in additional flowering from lateral branches. Based on grafting experiments^20^ it was hypothesised that the anticipation of flowering in the *Spcdc25* expressing plants may be result from an earlier competence of the shoot apical meristem to respond to the floral stimulus^15^. A similar mechanism may be operating in the tobacco plants expressing *Arath;WEE1*. It is also possible that *Arath;WEE1* tobacco plants have similar perturbations in cytokinin signalling and carbohydrate status that were noted in *Spcdc25* expressing tobacco plants^15,18,19^, although this would require further verification.

The reduction in primary root length and lateral root production in tobacco plants expressing *Arath;WEE1* contrasts with the effect of *Spcdc25* in increasing lateral root production noted by^26^. However, it is consistent with later reports of a restriction in root growth elicited by *Spcdc25* expression in tobacco and attributed to a replacement of cytokinin effects in the roots^15^. Shorter primary roots were also found when *Arath;WEE1* was over-expressed in Arabidopsis^24^ and is consistent with a negative effect of increased *WEE1* on root meristematic cell division.

The spontaneous formation of shoots in the absence of added cytokinins was also seen both in tobacco expressing *Spcdc25* and *Arath;WEE1*. However it contrasts with the phenotype seen in Arabidopsis plants over-expressing *Arath;WEE1* where cultured hypocotyls from the *Arath;WEE1* over-expressors produced fewer shoots than WT^24^. In fact the phenotype of the tobacco plants expressing *Arath;WEE1* in this respect is more similar to the *Arath;WEE1* knockout mutant lines, which produced more shoots from cultured hypocotyls than WT^24^.

Thus at a plant and organ level there are strong similarities between the effects of expressing *Spcdc25* and *Arath;WEE1* in tobacco. This is surprising given the opposing functions of the enzymes encoded. The difference between the expression of *Arath;WEE1* in tobacco and Arabidopsis confirms that *Arath;WEE1* does indeed induce the expected phenotype when expressed in its native environment. However the effects of its expression in tobacco are more consistent with a dominant negative effect, somehow repressing the action of the native *Nicta;WEE1*.

At a cellular level expression of *Arath;WEE1* also had a positive effect on cell division, very similar to that seen with the expression of *Spcdc25*^17^. This effect was independent of the insertion location or the construct since multiple tobacco BY2 lines of both constitutively expressed and inducible *Arath;WEE1* created multiple times in the lab all had the same phenotype. In most of the transgenic lines, the reduction in mitotic cell area in *Arath;WEE1* expressing tobacco BY2 cells was not quite as severe as that seen when *Spcdc25* was expressed, and indeed the *Arath;WEE1* expression did not induce the formation of double files of cells as was seen in the *Spcdc25* expressing cell lines^17^. However in one line, c-WEE1 line 10 where mitotic cell area was as low as seen in *Spcdc25* expressing lines, double files of cells were also visible. This indicates a threshold effect for the production of double cell files. As previously suggested^15^ the double cell files are reminiscent of the initial divisions in the pericycle that lead to the production of lateral root primordium. It is possible that the increase in lateral roots seen in some *Spcdc25* expressing tobacco plants may be related to the severity of the effect on meristematic cell size. The reduction in root mass and in lateral roots in *Spcdc25* and *Arath;WEE1* expressing plants may therefore be consistent with a less severe cellular phenotype when the transgene is expressed constitutively as was the case here and in Bell *et al*.^14^ as opposed to an inducible vector^26^.

Effects on cell cycle progression again were strikingly similar between BY2 cells expressing *Arath;WEE1* or *Spcdc25* with both showing a dramatic reduction in the length of the G2 phase and a lengthening of G1 + M phase^17^. In *Spcdc25*-expressing cells the anticipated mitotic peak was matched by an earlier increase in CDKB activity. Consistent with previous reports^4,5^, CDKB activity was also high at G2/M in uninduced cells. However, it peaked much earlier, in S phase in the cultures expressing *Arath;WEE1*. The anticipation of the mitotic peak when *Arath;WEE1* expression was induced, was also accompanied by a premature fall in WEE1 protein and kinase activity, consistent with the changes in WEE1 seen in WT cells^23^. Thus at a cellular level the induced *Arath;WEE1* expressing cell cultures are consistent with an early induction of mitosis after a short G2 resulting in a smaller mitotic cell size.

Expression of *Arath;WEE1* in tobacco BY2 cells resulted in the opposite phenotype to that found with *Solly;WEE1* expression in BY2 cells^25^ and indeed over-expression of *Nicta;WEE1* in the tobacco BY2 cells essentially had no effect. The results here also contrast with the effects on cell size seen when *Arath;WEE1* was over-expressed in Arabidopsis plants^24^ where root meristematic cells were larger than in WT.

One hypothesis to explain these unexpected results was that the expression of *Arath;WEE1* in the BY2 cells was causing an overall reduction of WEE1 protein perhaps due to a reduction of the native *Nicta;WEE1* transcript. However overall WEE1 protein was higher, and neither *Nicta;WEE1* or overall WEE1 transcript (*Arath;WEE1* + *Nicta;WEE1*) changed dramatically on induction of *Arath;WEE1* expression in exponentially growing BY2 cells. This indicates that the phenotypic effect is not due to a cell cycle-independent activation of the RNAi degradation pathway, which can be activated even with sense expression of transgenes^27^. A sense silencing mechanism is also less plausible given that in all three vector systems (BIN-HYG-TX^28^, pTA7002^29^ and pKanII-SPYCE^30^) used to express *Arath;WEE1* in BY2 cells the orientation of the constructs is such that read through of antisense transcript from the selectable marker construct is not possible. This was shown to be a key factor in sense-mediated silencing^27^.

However the apparent shift in the expression of the native *Nicta;WEE1* may form the underlying mechanism for the activation of a premature mitosis with the resulting phenotypic effects seen at a cellular, organ and whole plant level. In both WT and uninduced BY2 cells, *Nicta;WEE1* transcripts are most abundant during S phase. This is consistent with the slightly later accumulation of WEE1 protein during S + G2 phase. However, when *Arath;WEE1* is expressed, the peak of *Nicta;WEE1* transcripts in S phase seems to be replaced by a later expression peaking in M/G1. *Arath;WEE1* expression in these induced cultures is expressed more evenly through the cell cycle with a slight peak in S phase. This pattern is broadly consistent with reports on the expression of the 35S promoter during the cell cycle which show either a peak in S phase^28^ or constant expression throughout all phases^29^. One possible mechanism is that *Arath;WEE1* transcript production and translation into protein during S + G2 results in a feedback to *Nicta;WEE1* transcription, delaying the accumulation of native WEE1 transcripts. This could be mediated through the large number of transcription factors that are thought to regulate WEE1 expression that include *AtTCP15*^30^, *SOG1*^31^ and many others. An alternative mechanism may act at the protein level. The accumulation of Arath;WEE1 protein in S/G2 may activate the proteasome machinery prematurely due to differences in its sequence (Figs S3 and S4) and/ or conformation to trigger an early mitosis.

In conclusion the key finding is that expression of *Arath;WEE1* in tobacco causes an anomalous phenotype consistent with a dominant negative effect and a phenotype that strongly resembles expression of the positive regulator of G2/M progression, *Spcdc25*. This can be used as a useful tool to explore effects of down-regulating WEE1 action on plant development and cellular function. Furthermore, a full understanding of the underlying mechanism may throw light on the interaction of WEE1 with cellular machinery at a transcriptional and/or protein level.

## Materials and Methods

### WEE1 constructs

For expression of *Arath;WEE1* in BY2 cells the *Arath;WEE1* open reading frame was PCR amplified using primers P35SX (5′-AGGCCCCGGCTCGAGATGTTCGAGAAGAACGG-3′) and P36SS (5′GCACACTAGTCGACTCAACCTCGAATCCTAT-3′) and cloned into the BIN HYG TX vector^32^ under an attenuated form of the 35S promoter (as described in^24,33^) for constitutive expression, or into the inducible vector pTA7002^34^ using Xho I/Spe I. Individual clones were sequenced and a clone for each construct in which the amino acid sequence was intact was chosen for further work. For expression in whole tobacco plants, *Arath;WEE1* was cloned into pkanII-SPYCE(M)^35^ as described in Lentz Grønlund *et al*.^35,36^. *Nicta;WEE1* was cloned into the pTA7002 vector as described in Cook *et al*.^23^.

### Transformation of tobacco BY2 cells and induction of transgene in inducible lines

Stable transformation of tobacco (*Nicotiana tabacum*) BY2 cells was achieved using a modified version of the method described by^37^ with the addition of 20 µM acetosyringon (Sigma-Aldrich) during co-cultivation of the *Agrobacterium* (LBA4404) with the BY2 cells. Transformants were selected on solidified BY2 medium (0.8% agar) supplemented with 250 µg/ml Timentin and 80 µg/ml hygromycin. Calli were cultured in 50 ml BY2 medium, 250 µg/ml Timentin and 80 µg/ml hygromycin until stationary phase (1–3 weeks). Cultures were subjected to at least four rounds of sub culturing before being used in synchrony experiments.

Induction of WEE1 expression in BY2 cells carrying the pTA002 construct was achieved by addition of DEX (Sigma, UK) to a final concentration of between 1 µM and 100 µM. Induction of *Arath;WEE1* was achieved using 1 µM DEX, while for the *Nicta;WEE1* lines 30 and 100 µM were tested. DEX was added immediately following release from the aphidicolin block for synchronised cells, and three days after subculture for assays on exponentially growing cultures.

### Tobacco and arabidopsis plant transformation

Young leaves from *Nicotiana  tabacum* var Samsun plants grown in soil were surface sterilised in 5% hypochlorite solution containing 100 ul/l Triton X-100 for 5 min with gentle agitation. Leaves were rinsed three times in sterile distilled water and cut into 1 cm^2^ squares using a razor blade. Leaf squares were co-cultivated for 20–30 min in 100 ml of *Rhizobium radiobacter* (*Agrobacterium tumefaciens*) LBA4404 cell suspension (containing the WEE1 construct) at OD600 of 0.5 in 1 × MS medium in 140 mm diameter Petri dishes. Leaf squares were then transferred to shooting medium (1 × MS, 3% sucrose, 0.8% agar, NAA 0.1 ug/l, BAP 1ug/l). Following 48 h at 22 °C in the light, Leaf squares were then transferred to shooting medium including 50 ug/ml hygromycin and 200 ug/ml carbenicillin and incubation was continued for 4–6 weeks with a weekly subculture until calli and shoots were visible. Shoots were then excised and further cultured in rooting medium (1 × MS, 3% sucrose, 0.8% agar) to induce rooting. Plantlets were transferred to soil and grown to maturity. Expression of the transgene was analysed by PCR using primers AtWEE1fw (AGCTTGTCAGCTTTGCCT) and AtWEE1rv (TCAACCTCGAATCCTATCA). Two lines expressing the transgene (lines #2 and #8) were selected for further experiments.

### Analysis of tobacco plants

Wild type and transgenic tobacco plants were grown from seed in a growth chamber at 22/18 °C day/night thermoperiod with 16 hrs illumination (irradiance 435 W m^−2^), and a relative humidity 50–75% as described in^38^. The leaves were numbered from the base (1 oldest) and when the first flower bud emerged, the length of leaves without the petiole was measured and leaves above 10 cm in length were counted. The age of the plants is given as days of growth after sowing.

### Tobacco roots analysis

Sterilized tobacco seeds were sown on a square Petri dish containing MS medium (Murashige and Skoog Basal Salt Mixture, plant cell culture tested, Sigma-Aldrich, St. Louis, USA) containing 3% sucrose, 2 cm apart. After 21 days of cultivation at 25 °C with 16 h illumination with PFD (photon flux density) approximately 100 μmol m^−2^ s^−1^ (daylight fluorescent tubes; Osram, Wintherthur, Switzerland) as described in^38^. The length of the main root was measured and lateral roots counted semi-automatically with Smart Root software. For visualisation of root primordia the clearing method was used. The roots were fixed in acetone overnight and then fixed in phosphate buffer and mounted in 65% aqueous glycerol. They were observed with an Olympus BX51 microscope equipped with anvApogee U4000 digital camera.

### Organogenesis

Tobacco stem segments, 1 cm long, were placed onto MS medium (Murashige and Skoog Basal Salt Mixture, plant cell culture tested, Sigma-Aldrich, St. Louis, USA) containing 3% sucrose, or SIM (shoot inducing medium) consisting of MS medium with 3% sucrose, 0.1 mg/l NAA (naphthalene acetic acid), and 2 mg/l BAP (benzylaminopurine) as described in^38^. After 21 days of cultivation, the number of shoots and protruded shoot primordia were counted.

### Synchronisation, measurement of mitotic index and cell area of BY2 cells

BY2 cells were subcultured every 7 d and division was synchronized as previously described^39^. The mitotic index was measured at hourly intervals after removal of aphidicolin by scoring ≥300 Hoechst-stained cells per slide in random transects using fluorescence microscopy (Olympus BH2, UV, λ = 420 nm). Mitotic cell area was measured for approximately 300 cells per experiment.

### RT-PCR

RT-PCR was performed as described in^33^. Total RNA was extracted from BY2 cells using TRI reagent (Sigma Aldrich, Gillingham, UK) and residual genomic DNA was removed by DNase treatment (Ambion, Austin, Tex., USA). RNA (5 µg) was reacted with Superscript II reverse transcriptase (GIBCO, Paisley, UK). To study expression of *Arath;WEE1*, primers were designed which do not amplify the endogenous tobacco Wee1 gene (Nicta;WEE1): Arath;WEE1fw, and Arath;WEE1R: GTGCATCTCCTTCTTCTACT. Thermocycle conditions were: 35 cycles of 95 °C (1 min), 55 °C (1 min), 72 °C (1 min). Two sets of specific primers for Nicta;WEE1: (Nicta;WEE1F: 5′-CCAAATGGAGCTCTGTGACC and Nicta;WEE1R: 5′-CTCTTCGATCGGCTGGCTCTTA; NtWEE1F3: 5′-AGGGGTAGCTCATTTAGA and NtWEE1TOTR: 5′-TGGCAAAAGTAGCACCATCA) were used to analyse the expression of the endogenous tobacco WEE1 gene, *Nicta;WEE1* (Tm = 60 °C and 55 °C respectively). The first set was used for the quantification of *Nicta;WEE1* expression in synchronised cells while the second set were used to quantify expression in exponential phase cultures. For detection of *Nicta;WEE1* transgene expression only, a primer was designed to bind to the vector sequence: (35STRS 5′-ACGCTGAAGCTAGTCGACTC) and used in conjunction with NtWEE15R 5′-TTATCCCCATCGGCAGCATCAG. Histone H4 primers (H4F: 5′-GGCACAGGAAGGTTCTGAGGG ATAACA and H4R: 5′-TAACCGCCGAAACCGTAGAGAGTCC) were used to verify cell cycle stage, and primers to 18S rRNA: PUV2 5/-TTCCATGCTTAATGTATTCAGA and PUV4, 5/-ATGGTGGTGACGGGTGAC were used as a control^17^ (Tm = 60 °C). Thermocycler conditions were as above.

For all semi quantitative RT-PCR experiments, cycle number was reduced and optimised rigorously as described previously^24,40^ (Fig. S7) so that product amount was proportional to input amount of total RNA. This was verified with a dilution series of cDNA in each PCR experiment. Relative expression was normalised using primers to 18S rRNA as described previously^41^. A minimum of three replicate PCRs were performed for each primer set and products quantified from ethidium bromide stained agarose gels using the GeneGenius (Syngene, Cambridge, UK).

### Protein extraction, Western blotting and histone kinase assays

Proteins were extracted from Arabidopsis or tobacco leaves essentially as described in^42^. The WEE1 antibody and Western blotting were described in^35^. The antibody was used at a dilution of 1:1000 followed by α-rabbit IgG at 1:2500 (Sigma Dorset, UK). ECL reagents (Amersham Biosciences, Amersham, UK) were used to visualise the proteins.

For histone kinase assays proteins were extracted from 5 ml of synchronised cultures and assayed essentially as described in Cockcroft *et al*.^42^. Immunoprecipitations were carried out using antisera raised to Nicta;CDKA;1 and Nicta;CDKB1 as described in Sorrell *et al*.^4^. H1 protein kinase assays were as previously described^33,42^ using 2 µl of antiserum. Incorporation was assayed by quantitation of autoradiographs using the GeneGenius (Syngene, Cambridge, UK).

## Supplementary information

Supplementary Figures
