# Supplementary material for: Expression of Arabidopsis WEE1 in tobacco induces unexpected morphological and developmental changes
Source: Sci Rep. 2019 Jun 18;9:8695. doi: 10.1038/s41598-019-45015-3 (PMC6581958; doi:10.1038/s41598-019-45015-3)
Supplement: Supplementary file 1 — Supplementary Figures [file 41598_2019_45015_MOESM1_ESM.pdf]

Expression of Arabidopsis *WEE1* in tobacco, induces unexpected morphological and developmental changes.

Ilario Sicilano[a,c], Anne Lentz Grønlund [a], Hana Ševčíková[b], Natasha D. Spadafora[a,c], Golnaz Rafiei[a], Dennis Francis[a], Robert J. Herbert[c], M. Beatrice Bitonti[d], Hilary J. Rogers[a*], Helena Lipavská

Siciliano et al., Supp. Fig. 1

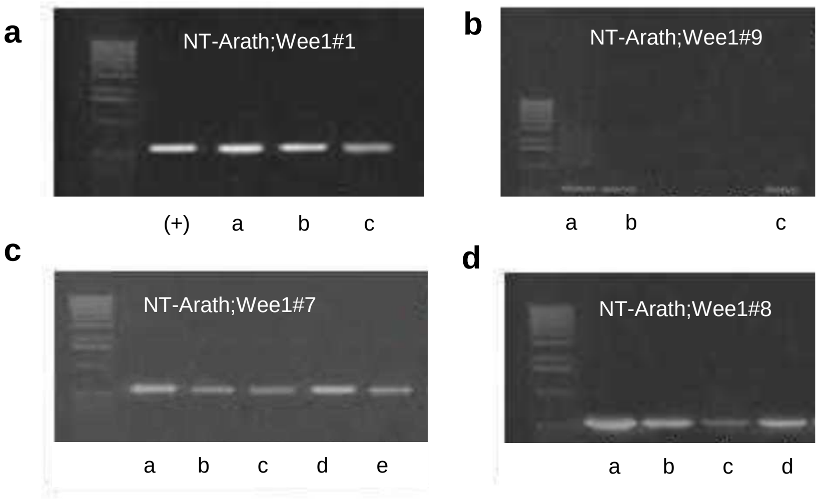

Supplementary Fig. 1: RT-PCR to confirm expression *Arath;Wee1 in* transgenic tobacco plants in the T2 generation (a) NT-Arath;Wee1#1 plants (+) = positive control (cDNA from Arabidopsis), a – c are individual plants (b) NT-Arath;Wee1#9 plants: a – c are individual plants (c) NT-Arath;Wee1#7 a – (d) NT-Arath;Wee1#8: a – d are individual plants

Siciliano et al., Supp. Fig. 2

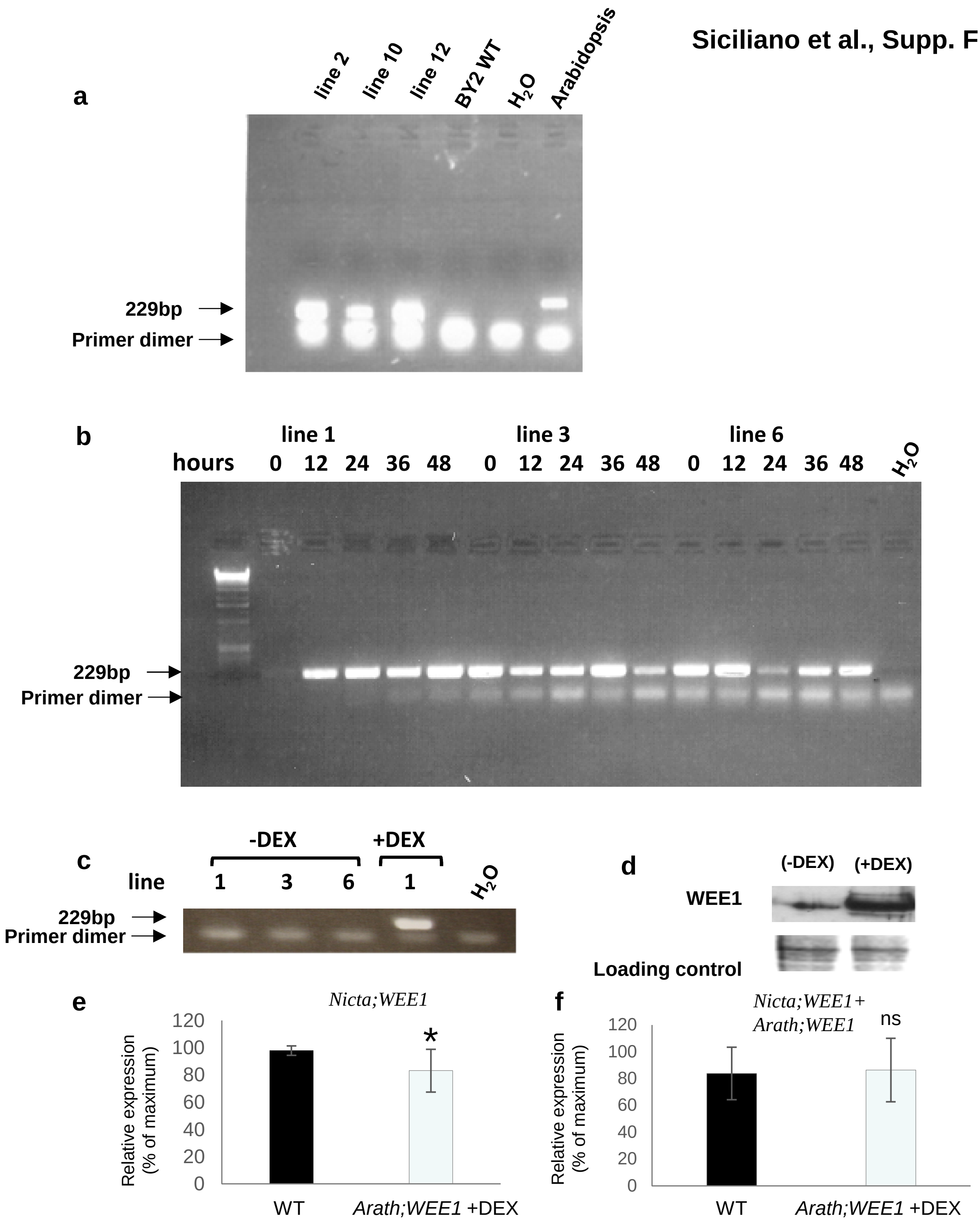

Supplementary Figure 2: Expression of WEE1 in unsynchronised tobacco BY2 cells (a) RT-PCR expression of *Arath;WEE1* in three independent BY-2 cell lines constitutively expressing *Arath;WEE1*: 2, 10 and 12 (b) RT-PCR profile of *Arath;WEE1* expression induced by dexamethasone (DEX) at intervals after induction in three independent lines: i-WEE1-1, 3 and 6 (c) comparison of induced (+DEX) and uninduced (-DEX) *Arath;WEE1* expression in *Arath;WEE1* inducible line i-WEE1-1 (d) WEE1 protein in BY2 cells expressing inducible *Arath;WEE1* ± induction with DEX. (e) Expression of *Nicta;WEE1* and (f) Expression of *Nicta;WEE1+ Arath;WEE1* in BY2 cell line expressing inducible *Arath;WEE1* induced with DEX, and WT BY2 cells; star indicates significant difference. (P <0.05) based on a Student's t test, ns= non-significant.

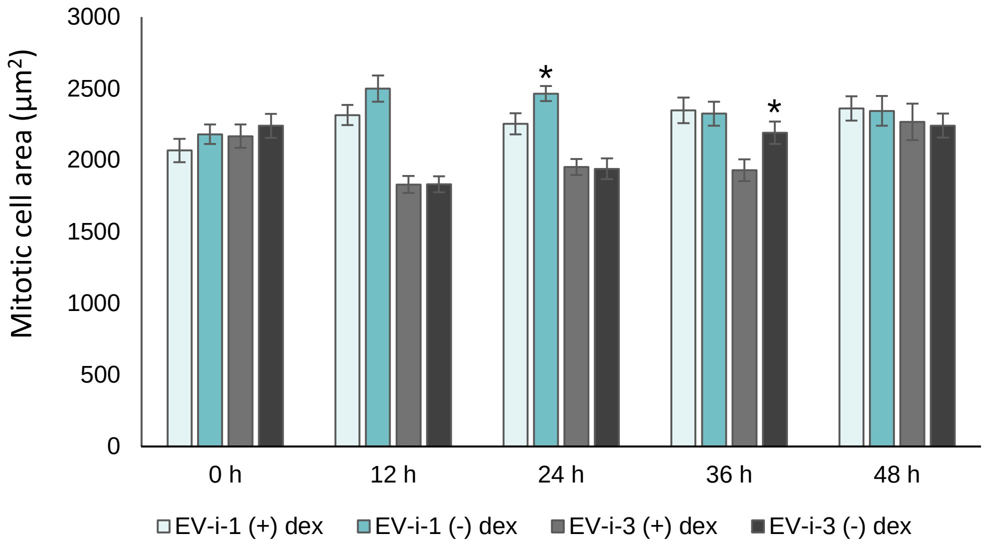

Supplementary Fig. 3: Effect of DEX on empty vector lines Mitotic cell area (µm^2) of two independent lines carrying the pTA7002 vector (empty vector, EV-i-1 and EV-i-3) +/- addition of dexamethasone (1 µM) in the first 48 hours following induction.

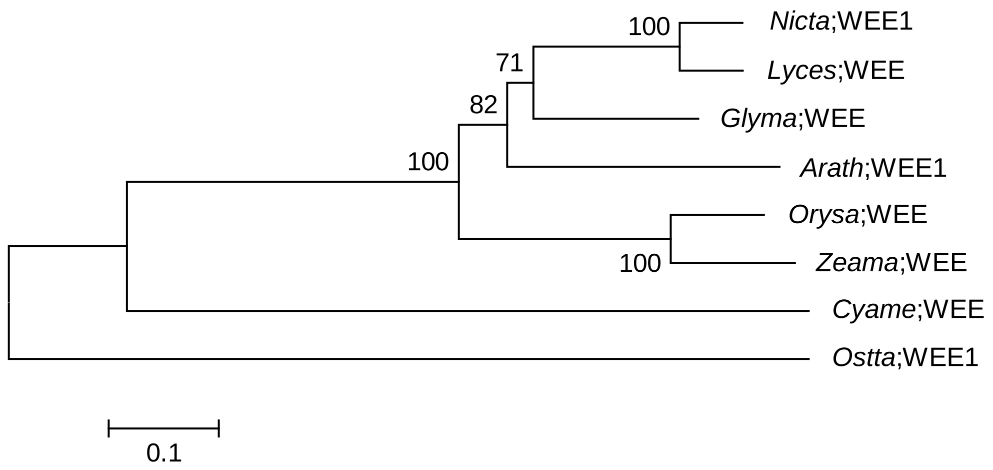

Supplementary Figure 4: phylogenetic tree showing relationship between five plant WEE1 protein sequences using MEGA3.0 (Kumar et al., 2004). The tree was constructed using the Neighbour-joining algorithm, bootstrap values are indicated at branches. *Zeama*;WEE1, *Zea Mays*, (AAD52983); *Arath*;WEE1, *Arabidopsis thaliana* (CAD28679) *Glyma*;WEE1, *Glycine max* (AAS13373), *Orysa*;WEE1, *Oryza sativa* (XP_464040); *Lyces*;WEE1, *Lycopersicon esculentum* (AM180939); *Cyame*;WEE1, *Cyanidioschyzon merolae* (CMT590C); *Ostta*;WEE1, *Ostreococcys tauri* (AY675101); *Nicta*;WEE1, *Nicotiana tabacum* (accession nos: AJ866274, AJ866275, AJ866276, and AJ866277)

Siciliano et al., Supp. Fig. 5

```
Nicta;WEE1      MKRKTLNRTSTPRRNNKSNTKRMNKGSLFT 30
Arath;WEE1      MFEKNG-RTLLAKRKTQGTIKTRASKKIRK 29

Nicta;WEE1      VGFSKVSLPPLPNQQQLQSSFSTLPLSN-- 58
Arath;WEE1      MEGTLERHSLLQFGQLSKISFENRPSSNVA 59

Nicta;WEE1      PSRFQKLLDSEVLPPAQSNFPSILPSNTDA 88
Arath;WEE1      SSAFQGLLDSD-----SSELRNQLGS-ADS 83

Nicta;WEE1      DAADGDNKDFILSQDFFCTPDYLTPDAPAI 118
Arath;WEE1      DANCGE-KDFILSQDFFCTPDYITPDNQNL 112

Nicta;WEE1      CNGLDGNKDDCMPCPKSPEKLQTVARKRQR 148
Arath;WEE1      MSGLDISKD-HSPCPRSPVKLNTVKSKRCR 141

Nicta;WEE1      LAVKSATSLSSDFPGQQQLADIPEDAFGSD 178
Arath;WEE1      QESFTGNHSNSTWSSKHRVDEQENDDIDTD 171

Nicta;WEE1      ETKSEKITESEKGHS-YVSQSAIALRYRVM 207
Arath;WEE1      EVMGDKLQANQTERTGYVSQAAVALRCRAM 201

Nicta;WEE1      PPPCIRNPYLRDASEIDVDPFGNRRSKCAG 237
Arath;WEE1      PPPCLKNPYVLNQSETATDPFGHQRSKCAS 231

                                             I
Nicta;WEE1      FNPVIFGNDGLSRYRSDFHEIEQIGTGNFS 267
Arath;WEE1      FLPVSTSGDGLSRYLTDFHEIRQIGAGHFS 261

                              II
Nicta;WEE1      RVFKVLKRIDGCMYAVKHSTKQLHQDTDRR 297
Arath;WEE1      RVFKVLKRMDGCLYAVKHSTRKLYLDSERR 291

                     III         IV
Nicta;WEE1      KALMEVQALAALGPHENVVGYYSSWFENEH 327
Arath;WEE1      KAMMEVQALAALGFHENIVGYYSSWFENEQ 321

                         V
Nicta;WEE1      LYIQMELCDHSLSN--KKYSKLSSEVAVLE 355
Arath;WEE1      LYIQLELCDHSLSALPKKSSLKVSEREILV 351

                       VIa        VIb
Nicta;WEE1      AMYQVAKALQYIHQRGVAHLDVKPDNIYVK 385
Arath;WEE1      IMHQIAKALHFVHEKGIAHLDVKPDNIYIK 381

                       VII              ▼▼▼   VIII
Nicta;WEE1      SGVYKLGDFGCATLLDKSQPIEEGDARYMP 415
Arath;WEE1      NGVCKLGDFGCATRLDKSLPVEEGDARYMP 411

                                  IX
Nicta;WEE1      QEILNENYDHLDKVDVFSLGAAIYELIRGS 445
Arath;WEE1      QEILNEDYEHLDKVDIFSLGVTVYELIKGS 441

                           X
Nicta;WEE1      PLPESGPHFLNLREGKLPLLPGHSLQFQNL 475
Arath;WEE1      PLTESRNQSLNIKEGKLPLLPGHSLQLQQL 471

                                          XI
Nicta;WEE1      LKVMMDPDPTRRPSAKDLVDNPIFERCQRN 505
Arath;WEE1      LKTMMDRDPKRRPSARELLDHPMFDRIRG- 500

Nicta;WEE1      ANK 508
Arath;WEE1      --- 500
```

Supplementary Figure 5:

Alignment of *Nicta;WEE1* to other plant WEE1 genes showing conserved (black) and similar (grey) residues. Roman numerals indicate kinase sub-domains in the catalytic domain. Closed circles indicate residues conserved in all the ORFs, inverted closed triangles indicate the conserved 'EGD' motif. Asterisks indicate two serine residues in Arath;WEE1 which conform to the RXXS consensus for phosphorylation sites and which are in similar positions to those in animal and yeast WEE1 proteins known to be involved in 14-3-3 regulation.

Siciliano et al., Supp. Fig. 6

Full blot image for Figure 6 (b)

-DEX

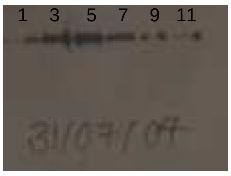

Full gel images for Figure 6 (b)

-DEX

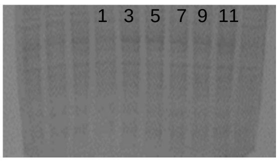

+DEX

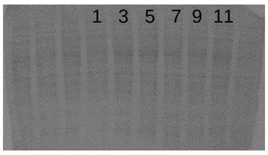

Full blot image for Supp. Fig 2(d)

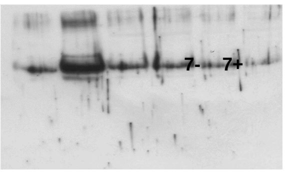

Full gel image for Supp. Fig 2(d)

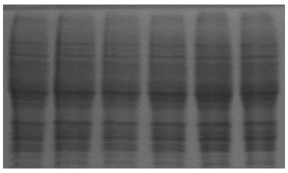

Supplementary Figure 6: Full gel images for blots and gels shown as sections in Figures and Supp Figures for the reviewers.

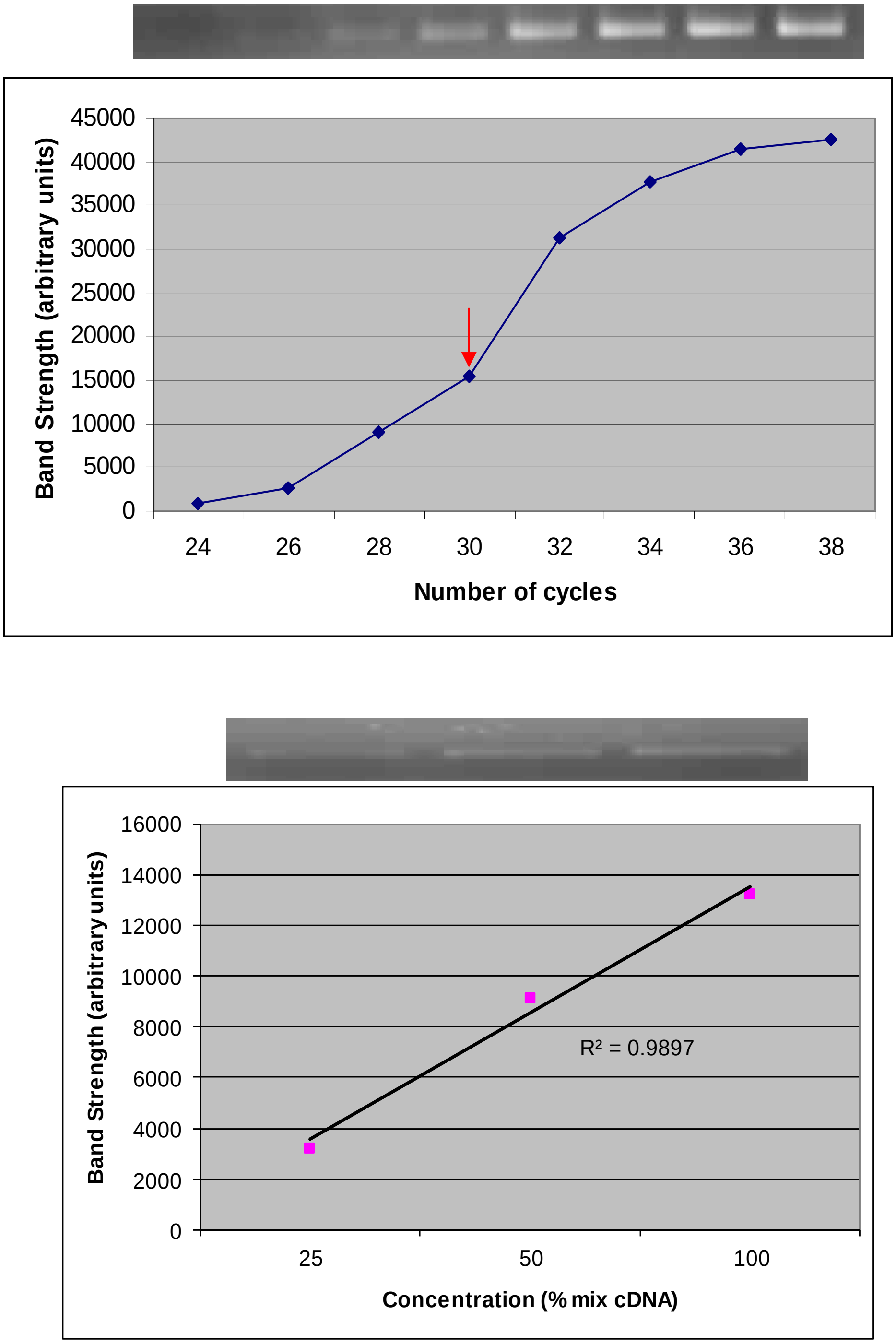

Supplementary Figure 7: Basis for semi-quantitative analysis of real-time PCR (a) Example of estimation of optimum cycle number to use for semi-quantitative RT-PCR for histone H4 from WEE1-i-1 cDNA. Arrow indicates cycle number chosen for further experiments**, (b)** Calibration plots using dilutions of WEE1-i-1 mix cDNA sample and histone H4 primers at an optimum number of cycles.

Siciliano et al., Supp. Fig. 8

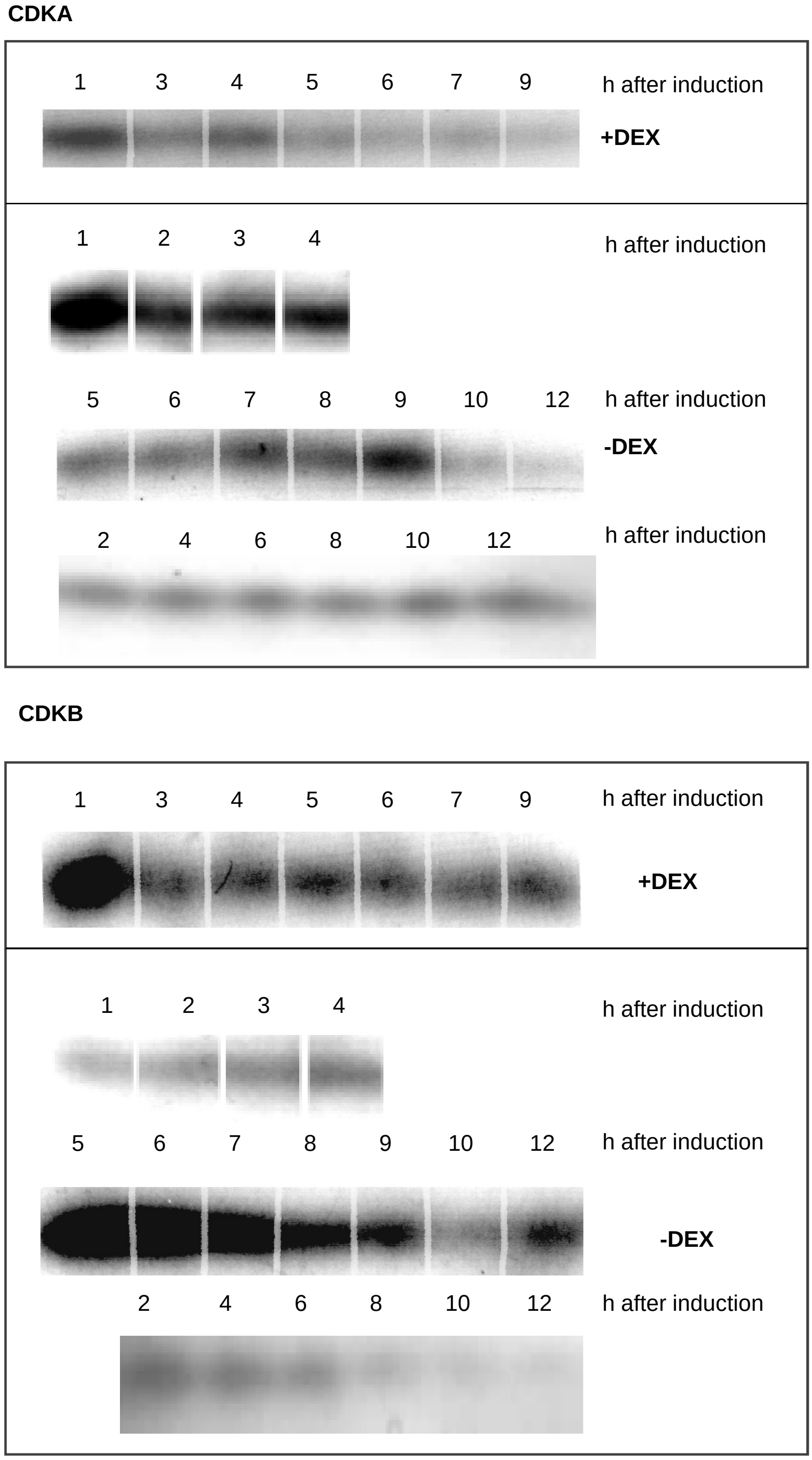

Supplementary Figure 8. Nicta;CDKB1 kinase activity is altered when *Arath;WEE1* is expressed in BY-2 cells CDKA and CDKB H1 kinase activities in i-WEE1-1, ± DEX. Immunoprecipitates from replicate experiments were quantified and shown as a % of maximum in Figure 7.

Note that early and later time points for the –DEX experiments were derived from separate gels and therefore absolute band intensity will vary amongst the gels. Hence they were normalised to % of maximum for deriving quantitative data.
